# Antioxidant vitamins supplementation reduce endometriosis related pelvic pain in humans: a systematic review and meta-analysis

**DOI:** 10.1186/s12958-023-01126-1

**Published:** 2023-08-29

**Authors:** Sai-Hua Zheng, Xiu-Xia Chen, Yi Chen, Zhi-Cong Wu, Xian-Qian Chen, Xue-Lian Li

**Affiliations:** 1 Gynaecology Department, The First Hospital of PuTian City, Putian, 351100 Fujian China; 2 Obstetrics and Gynecology Hospital, Fudan University, Shanghai, 200011 People’s Republic of China; 3 Shanghai Key Laboratory of Female Reproductive Endocrine Related Diseases, Obstetrics and Gynecology Hospital, Fudan University, Shanghai, 200011 People’s Republic of China; 4 Shanghai Clinical Research Center for Gynecological Diseases (22MC1940200), Shanghai Urogenital System Diseases Research Center (2022ZZ01012), Shanghai, 200011 People’s Republic of China

**Keywords:** Vitamin E, Vitamin C, Vitamin D, Antioxidant, Supplementation, Pain, Endometriosis

## Abstract

**Objective:**

This study aimed to clarify the effect of antioxidant vitamins supplementation on endometriosis-related pain.

**Methods:**

A systematic search of PubMed, Web of Science, Cochrane Library, Scopus, and China National Knowledge Infrastructure (CNK) databases was conducted to identify relevant studies published in English and Chinese up to 16 March 2023. The search terms used were "endometriosis" OR "endometrioma" OR "endometrium" AND "antioxidant" OR "Vitamin C" OR "Vitamin E" OR "Vitamin D" OR "25-OHD" OR "25(OH)D" OR "25-hydroxyvitamin D". Eligible studies were randomized controlled trials (RCTs) that assessed pain scores using the Visual Analogue Scale (VAS). Mean differences or odds ratios (ORs) with 95% confidence intervals (CIs) were calculated to evaluate the effect of antioxidant vitamins supplementation on endometriosis. The quality of the included studies was assessed using the Cochrane Risk of Bias Tool. The study was conducted following the Preferred Reporting Items for Systematic Reviews and Meta-analyses (PRISMA) guidelines.

**Results:**

A total of 13 RCTs involving 589 patients were included in this meta-analysis. We identified 11 studies that evaluated the effect of antioxidant vitamins supplementation on endometriosis-related pain. The results indicated that the supplementation of antioxidant vitamins can effectively alleviate endometriosis-related pain. Subgroup analysis showed that the supplementation of vitamin E (with or without vitamin C) had a positive effect on improving clinical pelvic pain in patients with chronic pelvic pain. Conversely, supplementation of vitamin D was associated with a reduction in pelvic pain in endometriosis patients, but the difference was not statistically significant compared to the placebo. Additionally, we observed changes in oxidative stress markers following vitamin supplementation. Plasma malondialdehyde (MDA) concentration decreased in patients with endometriosis after antioxidant vitamin supplementation, and the plasma MDA level was inversely correlated with the time and dose of vitamin E and C supplementation. Furthermore, the inflammatory markers in peritoneal fluid, including RANTES, interleukin-6, and monocyte chemoattractant protein-1, significantly decreased after antioxidant therapy. These findings suggest that antioxidant vitamins may alleviate pain in endometriosis patients by reducing inflammation.

**Conclusions:**

The included studies support the potential role of antioxidant vitamins in the management of endometriosis. Supplementation with antioxidant vitamins effectively reduced the severity of dysmenorrhea, improved dyspareunia and pelvic pain, and enhanced quality of life in these patients. Therefore, antioxidant vitamin therapy could be considered as an alternative treatment method, either alone or in combination with other approaches, for endometriosis-related pain.

**Trial registration:**

PROSPERO registration number: CRD42023415198.

**Supplementary Information:**

The online version contains supplementary material available at 10.1186/s12958-023-01126-1.

## Introduction

Endometriosis is a common gynecological disorder in reproductive-age women, characterized by a 10% incidence rate [1]. The condition leads to infertility and pain symptoms such as chronic pelvic pain, dysmenorrhoea, and deep dyspareunia [2, 3]. The impact of endometriosis on patients' quality of life is significant, affecting daily activities, sexual function, and personal relationships. Additionally, the disease is linked to depression, fatigue, and a decrease in work productivity, resulting in a substantial economic burden [4]. Studies estimate that endometriosis costs the US $78 billion annually in direct and indirect expenses, including healthcare resource utilization and lost productivity [5]. Despite its prevalence, there is currently no known cure for endometriosis, and the diagnosis is often delayed by 4 to 11 years from the onset of symptoms, even in developed countries [6].

The pathophysiology of endometriosis is not completely understood, although Sampson's retrograde menstruation theory is widely accepted [7]. However, the reason why some women with retrograde menstruation develop endometriosis while others do not remains unclear. Recent studies indicate that immunity and chronic inflammation play a role in its pathogenesis [8]. Peritoneal fluid analysis has shown increased levels of inflammatory cytokines, neutrophils, macrophages, and tumor necrosis factor-a [9, 10]. Notably, endometriosis is primarily associated with chronic pelvic pain resulting from macrophage and mast cell activation, contributing to a persistent cycle of inflammation, oxidative stress, and pain [11].

Oxidative stress, characterized by an imbalance between reactive oxygen species (ROS) and biological antioxidants, is believed to be a key factor in endometriosis pathophysiology [12]. Cells possess an antioxidant system to counteract the effects of ROS and maintain a balance between antioxidant defense and ROS production, thus preventing ROS-induced cellular damage and facilitating repair [13]. Therefore, increasing antioxidant levels may help reduce endometriosis-related pathology caused by oxidative damage [14].

Consequently, the effectiveness of antioxidant therapy in treating endometriosis has gained attention in recent years. A study by Jennifer et al. demonstrated that women with endometriosis showed improved peripheral antioxidant markers after adopting a high antioxidant diet [15]. Other studies have indicated that garlic extract can reduce pelvic and back pain, dysmenorrhea, and dyspareunia in women with endometriosis due to its ability to decrease oxidative stress, prostaglandin production, limit endometrial cell proliferation, and improve estrogen elimination [16, 17]. Furthermore, a higher intake of fruits, particularly citrus fruits, has been associated with a lower risk of endometriosis [18]. Vitamins supplementation has also shown promise in reducing endometriosis-related pelvic pain and improving the response to oxidative stress [12, 19–21]. Mounting evidence suggests a significant role of oxidative stress in endometriosis pathogenesis, with disease severity often corresponding with the levels of oxidative stress markers [12, 22].

Nevertheless, the data on the effects of antioxidant vitamins such as vitamin D, vitamin C, and vitamin E in countering oxidative stress in women with endometriosis is inconsistent. While one study by Almassinokiani et al. found that a 12-week supplementation of 50,000 IU vitamin D after ablative surgery for endometriosis did not significantly impact dysmenorrhea (*p* = 0.45) and pelvic pain (*p* = 0.24) [23], another study from Iran reported a significant improvement in dysmenorrhea (*p* = 0.03) with 12-week supplementation of 50,000 IU vitamin D given every two weeks, without affecting dyspareunia or dyschezia [24]. Therefore, the objective of this study is to provide a systematic review and meta-analysis to clarify the potential effects of antioxidant vitamins supplementation on endometriosis. This review aims to offer an overview of the current knowledge and assess future prospects related to new treatment options.

## Methods

### Search strategy

To conduct this study, a comprehensive search strategy was employed to locate relevant published studies. Electronic databases such as Web of Science, PubMed, Cochrane Library, Scopus, and China National Knowledge Infrastructure (CNKI) were searched from inception up to 16 March 2023. The search keywords used included "endometriosis" OR "endometrioma" OR "endometrium" AND "antioxidant" OR "Vitamin" OR "Vitamin C" OR "Vitamin E" OR "Vitamin D" OR "25-OHD" OR "25(OH)D" OR "25-hydroxyvitaminD". Additionally, the reference lists of relevant studies were also examined to minimize any potential omissions. Both English and Chinese articles were included in this research. The details of the search strategy can be found in Supplementary Data File S1.

### Inclusion and exclusion criteria

Inclusion and exclusion criteria were established based on the PICOS (Population/patients, Intervention/exposure, Control/comparison, Outcome, and Study type) strategy, as presented in Table 1. Only studies meeting these criteria were included in the analysis.

**Table 1:** Formulated question of the study based on PICOS

Inclusion criteria	
P(Participants)	Endometriosis patient
I(Intervention)	Vitamin C/E/D with no limits on the dose,frequency and so on
C(Comparisons)	No supplementation or placebo
O(Outcomes)	Primary:chronic pelvic pain,dysmenorrhea,dyspareunia; Secondary outcome: oxidative stress markers,fertility
S(Study type)	Randomized controlled trials(RCTs)

Our exclusion criteria were as follows: (1) Studies without accessible full-text; (2) Comments, reviews, case series, letters, in vitro studies, and editorials; (3) Incomplete original initial literature data.Our study protocol was registered online through PROSPERO (CRD42023415198). We followed the Preferred Reporting Items for Systematic Reviews and Meta-analyses (PRISMA) guidelines to complete the study. The structures of the articles we searched were appraised using the reconstructed PRISMA checklist (Supplementary Data File 2).

### Study selection and data extraction

The two researchers independently conducted full-text reviews and selected literature based on inclusion/exclusion criteria by title and abstract. Duplicate studies were excluded. One author extracted the data while another examined it, resolving inconsistencies through discussion and involving third party researchers when necessary. The collected data included information such as first author, year of publication, country, study sample size, type of study, age, dose and duration of antioxidant supplementation, and early morning results. For each Randomized Controlled Trial (RCT) included, information regarding randomization, allocation, blinding, reproducibility, relevancy, and outcomes were collected. Both authors critically analyzed the results and used the GRADE System to rate the quality of the evidence and grade the strength of the recommendation for each result.

### Risk of bias assessment

The quality of the trials was assessed independently by two reviewers, and any discrepancies were resolved through consensus. The assessment of the risk-of-bias of RCTs was done according to the Cochrane Handbook for the Development of Systematic Reviews of Intervention (Version 5.4.1), using the Cochrane Risk of Bias Tool consisting of six domains: selection bias, performance bias, attrition bias, detection bias, reporting bias, and other sources of bias. Each item was classified as low, high, or unclear risk of bias.

### Statistical analysis

Statistical analyses were performed using the comprehensive meta-analysis software (RevMan 5.4.1; Cochrane collaboration, the Nordic Cochrane Centre, Copenhagen, Denmark) and Stata 14. Pain scores, reported on different scales across studies, were presented as a standardized mean difference (SMD). For dichotomous variables, results were presented as odds ratios (ORs). Standardized mean difference (SMD) and their respective 95% confidence intervals (95% CI) were used for continuous outcome variables. The standard deviation (SD) of the change from baseline in the experimental intervention groups was calculated using the following equation, in which R_1_ = 0.5 [25]:

$$\mathrm{SD}(\mathrm{C})=\sqrt{{\mathrm{SD}(\mathrm{A})}^{2}+\mathrm{SD}{(\mathrm{B})}^{2}-(2\times \mathrm{R}1\times \mathrm{SD}(\mathrm{A})\times \mathrm{SD}(\mathrm{B})}$$

where SD(A) represents the standard deviation before intervention and SD(B) denotes the standard deviation after intervention. All data were analyzed using the chi-square test, with *p*-values < 0.05 considered statistically significant. Statistical heterogeneity was assessed using the I^2^ statistic and the Cochran's Q test, with a *p*-value < 0.1 indicating significant heterogeneity. When heterogeneity was significant (I^2^ > 50%), a random effect model based on the generic inverse variance method was used [26, 27]. Further exploration of statistical heterogeneity was done by calculating the prediction interval with each study omitted in turn [28]. Publication bias was assessed using funnel plots and Egger's tests.

## Results

### Study selection and characteristics

**Fig. 1:**
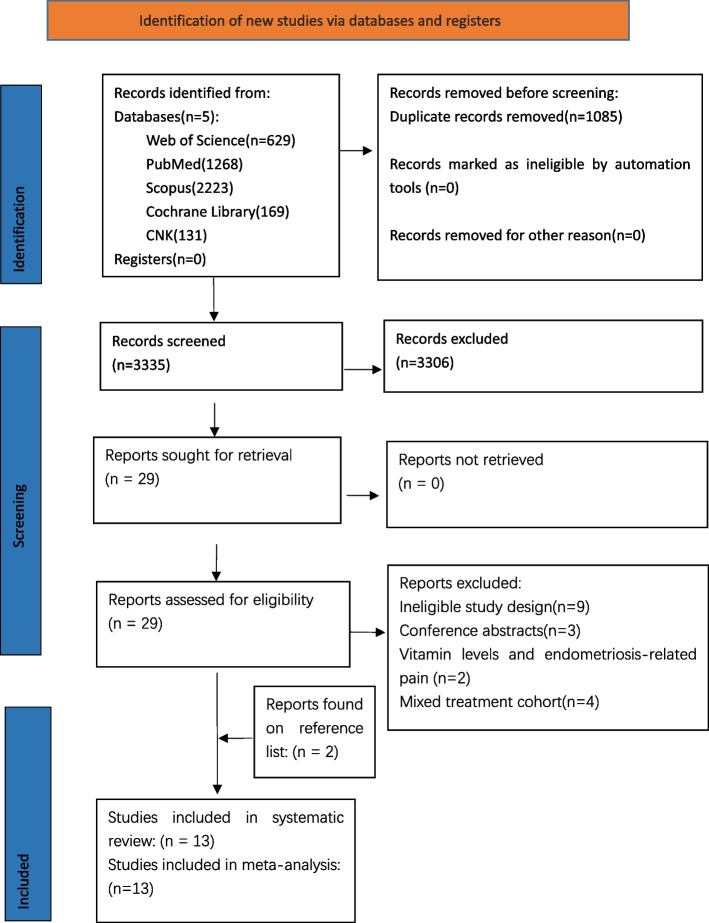
PRISMA flow chart for study identification and inclusion/exclusion

The electronic database search yielded 4420 articles, of which 1085 were duplicates. After filtering based on titles and abstracts, 3306 articles were excluded, leaving 13 studies eligible for inclusion, as detailed in the PRISMA flowchart in Fig. 1. These studies originated from Iran (3), China (4), Mexico (2), Germany (1), Egypt (1), the USA (1), and Atlanta (1). Among the included studies, 10 investigated the impact of vitamin supplementation on endometriosis-related pain, while 6 reported changes in oxidative stress markers after vitamin supplementation, and only 2 had available data on the impact of vitamin supplementation on pregnancy outcomes in women with endometriosis. The individual studies, their characteristics, and outcomes are described in Tables 2, 3, and 4 (provide reference to specific figures and tables if applicable).

**Table 2:** Study characteristics of 11 studies about Vitamin supplementation impact on endometriosis-related pain

Study	Country	Study type	Sample size (intervention/control)	Age(Mea ± SD)	Type and duration of supplementation		Primary outcomes		
					Intervention group	Placebo group	Chronic pelvic pain	Dysmenorrhea	Dyspareunia
Nalini Santanam et al.,2013 [29]	Atlanta	RCT	(46/13)	19-41year	vitaminE1200IU + vitamin C1000mg for 8 weeks	placebo pills daily for 8 weeks	Intervention group patient with decreased pain (43%) Control group patient with decreased pain (0%)	Intervention group patient with decreased pain (37%) Control group patient with decreased pain (36%)	Intervention group patient with decreased pain (24%) Control group patient with decreased pain(0%)
Ibrahim Abd El-Fadil Sehsah et al.;2022 [30]	Egypt	RCT	(50/50)	Intervention: 25.36 ± 3.75; Control: 26.18 ± 4.24	vitamin E 1200mg/day and vitamin C 1000mg/day for 6–8 weeks	placebo for 6–8 weeks	Intervention group patient with decreased pain (48%) Control group patient with decreased pain(10%)	Intervention group patient with decreased pain (48%) Control group patient with decreased pain(10%)	Intervention group patient with decreased pain (32%) Control group patient with decreased pain(8%)
Mahmoud A. Al-Naggar et al.;2022 [31]	Iran	RCT	(30/30)	Intervention: 32.5 ± 4.5 Control: 31.4 ± 5.2	vitaminE1200IU/day + Vitamin C 1000mg/day for 8 weeks	Placebo pills for 8 weeks	Intervention group patient with decreased pain (30%) Control group patient with decreased pain(0%)	Intervention group patient with decreased pain(26.6%) Control group patient with decreased pain(0%)	Intervention group patient with decreased pain (16.6%) Control group patient with decreased pain(0%)
Leila Amini et al,2021 [32]	Iran	RCT	(30/30)	Intervention: 35.7 ± 5.71; Control: 38.03 ± 6.47	vitaminE800IU + vitaminC1000mg for 8 weeks	placebo pills daily for 8 weeks	Intervention VAS score: pre-post: (66.26 ± 27.84– 12.43 ± 13.28) Control group VAS score :pre-post: (16.96 ± 16.28– 18.63 ± 18.35);P < 0.001	Intervention VAS score: pre-post: (50.53 ± 32.12–17.56 ± 16.65) Control group VAS scorec: pre-post: (51.00 ± 34.21– 31.56 ± 26.39);P < 0.001	Intervention VAS score pre-post: (66.26 ± 28.27– 15.43 ± 18.47) Control group VAS score: pre-post: (20.73 ± 21.77– 18.10 ± 19.93);P < 0.001
HuiMing Wang 2017 [33]	China	RCT	(32/32)	Intervention: 37.9 ± 3.5; Control: 35.0 ± 3.3	Yasmin + vitamin E 100mg/d for 6mo	Yasmin for 6mo	Intervention VAS score: pre-post: 7.85 ± 2.17- 2.65 ± 0.81; Control group VAS score: pre-post:7.90 ± 1.19—4.35 ± 1.11	Intervention VAS score: pre-post:6.75 ± 1.25–2.50 ± 1.00; Control group VAS score: pre-post:6.63 ± 1.27 –2.79 ± 1.21	Intervention VAS score: pre-post: 7.73 ± 2.15–3.01 ± 1.11; Control group VAS score: pre-post:7.65 ± 1.20–3.13 ± 1.01
Xiao Jing et al. 2018 [34]	China	RCT	(30/30)	Intervention: 35.12 ± 3.2; Control: 38.02 ± 4.01	Yasmin + vitamin E 100mg/d for 6mo	Yasmin for 6mo	Intervention VAS score: pre-post:7.83 ± 2.16–2.66 ± 0.82; Control group VAS score: pre-post: 7.81 ± 0.82–4.33 ± 1.10	Intervention VAS score: pre-post:6.77 ± 1.24–2.51 ± 1.02; Control group VAS score: pre-post: 6.62 ± 1.28–2.80 ± 1.20	Intervention VAS score: pre-post:7.72 ± 2.14–3.00 ± 1.12; Control group VAS score: pre-post: 7.66 ± 1.21–3.12 ± 1.02
Hu Yan et al. 2015 [35]	China	RCT	(26/25)	Intervention: 32.45 ± 6.18; Control: 34.62 ± 5.47	Yasmin + vitamin E 100mg/d for 6mo	Yasmin for 6mo	Intervention VAS score: pre-post:74.05(16.17)-30.14(15.29); Control group VAS score: pre-post:76.11(17.84)-51.37(13.54)	Intervention VAS score: pre-post:77.27(16.46) –42.33(10.15); Control group VAS score: pre-post:75.28(16.54)–44.08(12.80)	Intervention VAS score: pre-post:68.36(19.08)-50.41(13.78); Control group VAS score: pre-post:65.61(17.14)-49.24(12.63)
Fariba Almassinokiani et al.; 2016 [23]	Iran	RCT	(19/19)	Intervention:30.84 ± 5.79 Control: 28.95 ± 4.71	vitamin D 3(50 000 IU weekly for 12 weeks	placebo for 12 weeks	Intervention VAS score: pre-post:4.05 (3.45)–0.84 (1.74); Control group VAS score: pre-post: 4.82 (4.1) –0.68 (1.70)	Intervention VAS score: pre-post: 7.37 (2.61)–2.10 (2.33); Control group VAS score: pre-post:6.42 (3.04)–2.73 (2.84)	
Abolfazl Mehdizadehkashi et al.;2021 [24]	Germany	RCT	(25/25)	Intervention: 34.8 ± 7.1; Control: 35.6 ± 7.0	50,000 IU vitamin D every 2 weeks for 12 weeks	placebo for 12 weeks		Intervention VAS score: pre-post: (8.0 ± 1.8– 4.8 ± 1.7) Control group VAS score: pre-post: ( 6.8 ± 2.6– 4.9 ± 2.8);	Intervention VAS score: pre-post: ( 6.1 ± 2.6–3.8 ± 2.3) Control group VAS score: pre-post: (7.3 ± 2.5– 5.2 ± 2.6);
James L Nodler et al.; 2020 [36]	America	RCT	(27/20)	Intervention: 20.0 ± 2.7; Control: 20.1 ± 3.5	2000 IU vitamin D3 for 6mo	placebo for 6 mo	Intervention VAS score: pre-post:7.0 (2.2)– 5.5(3.1); Control group VAS score: pre-post 6.0 (1.9)- 4.4 (3.1)

**Table 3:** Study characteristics of 6 studies about Vitamin supplementation impact on oxidative stress markers in women with endometriosis

Study	Country	Study type	Sample size(intervention/control)	Age(Mean ± SD)	Type and duration of supplementation		Malondialdehyde(MDA) umol/L	Other oxidative stress markers
					Intervention group	Placebo group		
Jennifer Mier-Cabrera et al;2007 [37]	Mexican	RCT	(16/18)	Intervention: 32.7 ± 2.36 Control: 32.8 ± 2.49	vitaminE84mg + vitaminC343mg for 6	Placebo pills for 6weeks	Intervention:pre-post: ( 28.05 ± 3.97–11.47 ± 4.47); Control:pre-post: (27.69 ± 7.56–- 23.83 ± 8.10)	lipid hydroperoxides (LOHS) ↓,*P* < 0.05↑
Leila Amini et al, 2021 [32]	Iran	RCT	(30/30)	Intervention: 35.7 ± 5.71; Control: 38.03 ± 6.47	vitaminE800IU + vitaminC1000mg for 8 weeks	placebo pills daily for 8 weeks	Intervention:pre-post: (41 ± 2.37–17.74 ± 16.66); Control:pre-post: (32.09 ± 22.84–34.51 ± 28.66)	reactive oxygen spices (ROS)↓,*P* < 0.05, total antioxidant capacity unaffected
Abolfazl Mehdizadehkashi et al.;2021 [24]	Germany	RCT	(25/25)	Intervention: 34.8 ± 7.1; Control: 35.6 ± 7.0	50,000 IU vitamin D every 2 weeks for 12 weeks	placebo for 12 weeks	Intervention:pre-post: ( 6.1 ± 2.6–3.8 ± 2.3) Control:pre-post: (7.3 ± 2.5– 5.2 ± 2.6)	total antioxidant capacity(TCA)↓,*P* < 0.05;high-sensitivity C-reactive protein (hs-CRP) *P* < 0.05
Nalini Santanam et al.,2013 [29]	Atlanta	RCT	(46/13)	19-41year	vitaminE1200IU + vitamin C1000mg for 8 weeks			peritoneal fluid inflammatory markers↓, RANTES (*p* ≤ 0.002)↓ interleukin-6↓(*p* ≤ 0.056) and monocyte chemotactic protein-1 ↓ (*p* ≤ 0.016)
Jennifer Mier-Cabrera et al.,2009 [15]	Mexican	RCT	(37/35)	Intervention: 32.70 ± 2.46 Control:33.8 ± 3.15	high antioxidant diet (include f vitamin C 500 mg and vitamin E 20 mg) for 3mo	normal diet	Intervention:pre-post: ( 31.1 ± 4.7–23.0 ± 2.3) Control group:pre-post: (31.6 ± 5.4– 30.9 ± 3.2)	Lipid Hydroperoxides(LPH) ↓,*p* < 0.001
Xiang Lu et al.,2018 [38]	China	RCT	137/108	Intervention: 31.5 ± 3.5 Control:31.9 ± 3.00	Vitamin C 1000 mg/ day of 2mo	Non-treatment		oxidative stress markers were unaffected

**Table 4:** Study characteristics of 2 studies about Vitamin supplementation impact on fertility

Study	Country	Study type	Sample size(intervention/control)	Age(Mean ± SD)	Type and duration of supplementation		outcome
					Intervention group	Placebo group	
Xiang Lu et al.,2018	China	RCT	137/108	Intervention: 31.5 ± 3.5 Control:31.9 ± 3.00	Vitamin C 1000 mg/ day of 2mo	Non-treatment	retrieved oocyte rate, implantation rate, and clinical pregnancy rate unaffected;but ameliorating the quality of oocytes and embryos
Jennifer Mier-Cabrera et al;2007 [37]	Mexico	RCT	(16/18)	Intervention: 32.7 ± 2.36 Control: 32.8 ± 2.49	vitaminE84mg + vitaminC343mg for 6	Placebo pills for 6weeks	the pregnancy rate unaffected

### Primary outcome

#### Relationship between vitamin supplementation and endometriosis-related chronic pelvic pain

**Fig. 2:**
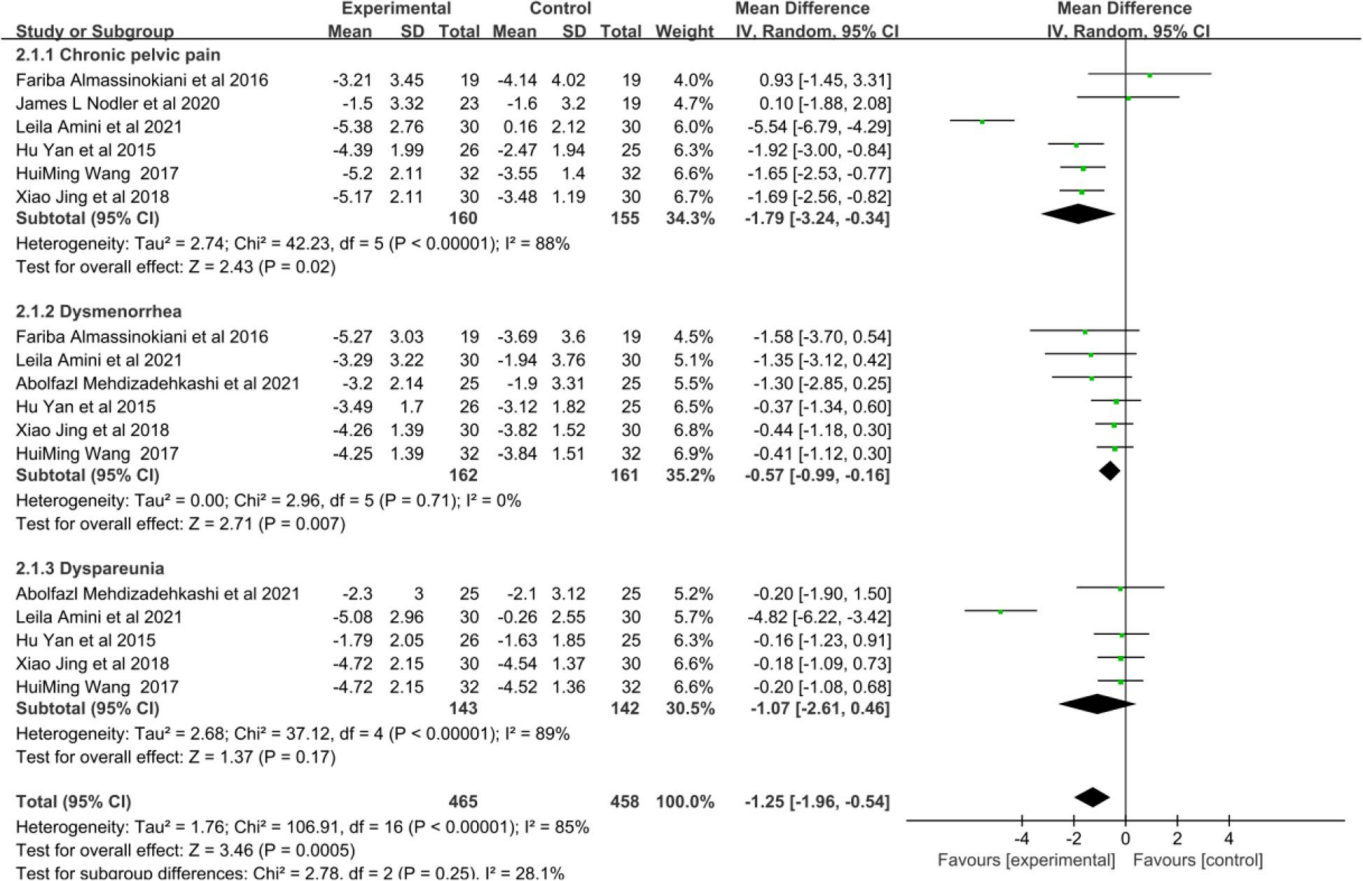
Forest plot (random effects model). for the VAS score, antioxidant vitamins supplementation and endometriosis related pain

**Fig. 3:**
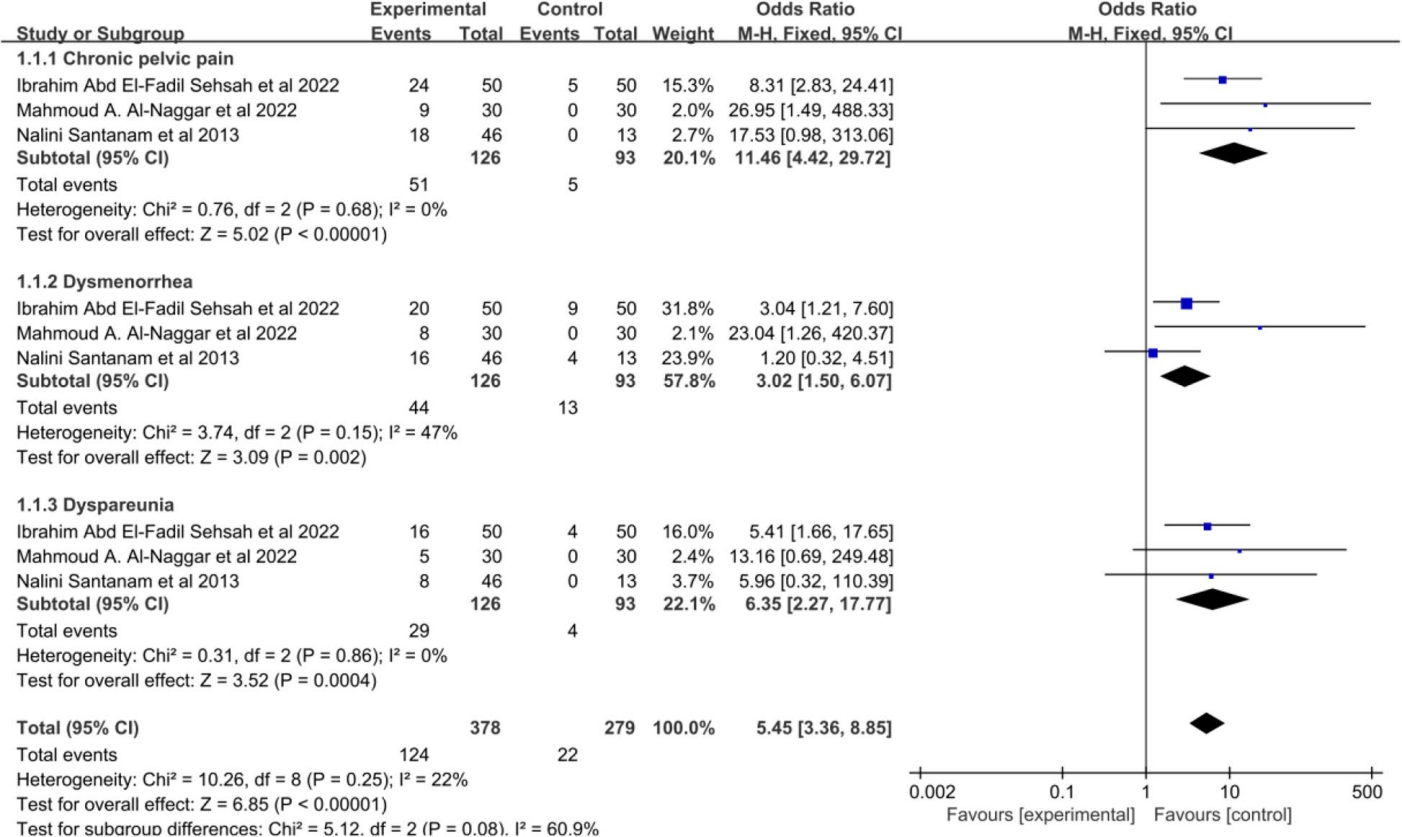
Forest plot (fixed effects model). for numbers of patients with decreased pain scores, antioxidant vitamins supplementation and endometriosis related pain

**Fig. 4:**
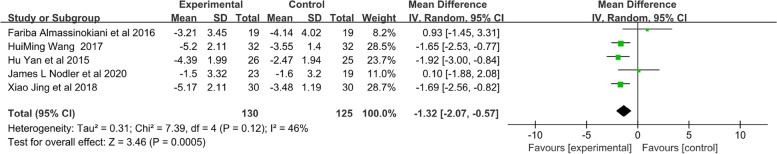
Forest plot (random effects model). for the VAS score, antioxidant vitamins supplementation and endometriosis related pain, when the Leila Amini et al. (2007) study was excluded

In our systematic review, a total of 534 patients were included in the 9 studies that analyzed the relationship between vitamins supplementation and endometriosis-related chronic pelvic pain. Among these studies, four of them used vitamins E and C as experimental groups with a placebo as the control group. The doses and durations of these studies were similar. Additionally, three randomized controlled trials examined the effects of vitamin E and oral contraceptives (Yasmin) on chronic pelvic pain, with the same doses and follow-up time. Two of the RCTs used vitamin D as intervention groups, with one study using a dosage of 50,000IU weekly for 12 months and the other using 2000 IU daily for 6 months. Furthermore, all of the RCTs evaluated their pain scores using the Visual Analogue Scale (VAS). Out of the six RCTs that reported pain scores before and after treatment in both groups, three studies reported the number of patients with decreased pain scores in both groups, specifically the Vitamin E and C groups versus the placebo group. The results of the meta-analysis indicated that vitamin supplementation significantly reduced chronic pelvic pain in endometriosis patients. For continuous data, the mean was -1.79 (95% CI: -3.24 to -0.34, *p* = 0.02, I^2^ = 88%, random effects model, Fig. 2), while for categorical data, the odds ratio was 11.46 (95% CI: 4.42 to 29.72, *p* < 0.00001, I^2^ = 0%, fixed effects model, Fig. 3). The heterogeneity between studies was high (I^2^ = 88%). Subgroup and sensitivity analyses were performed (Supplementary table 1), and at the subgroup level, both continuous and categorical data showed that vitamin E supplementation, with or without vitamin C, had a positive effect on improving clinical pelvic pain (mean = -2.65, 95% CI: -4.25 to -1.06, *p* = 0.0001, I^2^ = 88%, random effects model and OR = 11.46, 95% CI: 4.42 to 29.72, *p* < 0.00001, I^2^ = 0%, fixed effects model). On the other hand, vitamin D supplementation did show a reduction in pelvic pain in endometriosis patients, but it was not significant when compared to placebo (mean = 0.44, 95% CI: -0.18 to 1.96, *p* = 0.57, I^2^ = 0%, fixed effects model). When each study was sequentially omitted in an analysis (Supplementary table 2), the study by Leila Amini et al. (2007) contributed the greatest heterogeneity, and with this study omitted, the heterogeneity decreased to 49% with a narrower 95% CI prediction interval. The results of the meta-analysis remained unchanged (Fig. 4). The quality of the evidence was low according to GRADE (Supplementary Data File 3).

#### Relationship between vitamin supplementation and endometriosis-related dysmenorrhea

In terms of dysmenorrhea in endometriosis, our systematic review included 9 out of the 13 randomized controlled trials, which involved 544 women with endometriosis. Four of these trials used vitamins E and C as the experimental group, with a placebo as the control group. Three studies used oral contraceptives (Yasmin) in combination with vitamin E as the experimental group, and oral contraceptives alone as the control group. Their doses and follow-up time were the same. The other two studies used vitamin D as the experimental group, with placebo as the control group. In one of these studies, the dosage of vitamin D was 50,000 IU weekly for 12 months, while the other study used a dosage of 50,000 IU every 2 weeks for 12 months. Our systematic review and meta-analysis showed that all studies demonstrated the significant effectiveness of various vitamin supplementation in reducing dysmenorrhea (mean = -0.57, 95% CI: -0.99 to -0.16, p = 0.007, I^2^ = 0%, random effects model, Fig. 2 and OR:3.02, 95% CI: 1.50 to 6.07, *p* = 0.002, I^2^ = 0%, fixed effects model, Fig. 3). There was no heterogeneity among these studies (*p* = 0.71, I^2^ = 0%). The quality of the evidence was moderate according to GRADE (Supplementary Data File 3).

#### Relationship between vitamin supplementation and endometriosis-related dyspareunia

Among the 13 studies, 8 reported on endometriosis-related dyspareunia. However, the effects of different vitamin supplements on dyspareunia pain in patients with endometriosis were inconsistent. Subgroup analyses showed that patients receiving combined vitamin E and C supplement had significantly higher dyspareunia pain relief rates compared to the control group (OR: 6.35, 95% CI: 2.27 to 17.77, 3 studies, 219 patients, I^2^ = 0, fixed effects model, Fig. 3). The study by Leila Amini et al. (2007) also showed a significant improvement in dyspareunia pain with combined vitamin E and C supplementation for 8 weeks (mean: -4.82, 95% CI: -6.22 to -3.42, *p* = 0.0003). On the other hand, the quantitative synthesis of the included studies showed no difference between placebo and vitamin D or vitamin E supplementation alone for dyspareunia pain (mean: -0.18, 95% CI: -0.70 to 0.34, 4 studies, 225 patients, I^2^ = 0%, fixed effects model, Fig. 2). The quality of the evidence was low according to GRADE (Supplementary Data File 3).

### Secondary outcome

#### The effect of vitamin supplementation on peripheral oxidative stress markers in endometriosis

**Fig. 5:**
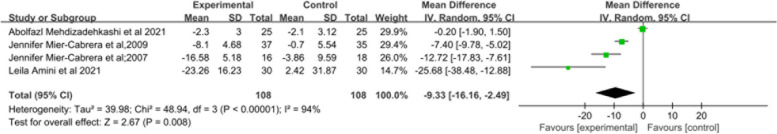
Forest plot (random effects model), the plasma MDA concentration and antioxidant vitamins supplementation in women with endometriosis

**Fig. 6:**
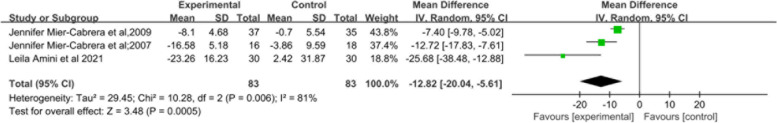
Forest plot (random effects model), the plasma MDA concentration and antioxidant vitamins supplementation in women with endometriosis, when Abolfazl Mehdizadehkashi et al. (2021) study was excluded

The details of the included studies are shown in Table 3. In the meta-analysis, five studies (*n* = 275) evaluated the effect of vitamin supplements on peripheral oxidative stress markers in women with endometriosis. Four of these studies used vitamin E + C as the vitamin supplement, while one study used vitamin D as the experimental group. Four randomized controlled trials (RCTs) reported the peripheral blood value of Malondialdehyde (MDA) before and after treatment in both groups. The results indicated that patients showed a reduction in MDA levels compared to the controls (mean: -9.33, 95% CI: -16.16 to -2.49, *P* = 0.008, I^2^ = 94%, random effects model, Fig. 5). There was high heterogeneity between the studies (I^2^ = 94%). When one study about vitamin D supplementation was removed, and the estimate was recalculated with the remaining studies on combined vitamin E and C supplementation, the result suggested an even greater reduction in MDA levels (mean: -12.82, 95% CI: -20.04 to -5.61, *P* = 0.006, I^2^ = 81%, random effects model, Fig. 6). Furthermore, an analysis of the dose and duration of vitamins E and C treatment revealed an inverse proportional relationship between plasma MDA concentration and the duration and dose of vitamin E and C supplementation. This indicates that the plasma MDA concentration decreased more significantly with increased vitamin intake. Therefore, the dose and duration of treatment may be the main potential source of heterogeneity.

Similarly, compared with the control group, other oxidative stress markers also showed a significant decrease with statistical significance (Table 3). Two studies demonstrated lower significant levels of lipid hydroperoxides in the experimental group (*P* < 0.05) [15, 39]. Leila Amini et al. found a significant reduction in reactive oxygen species (ROS) compared with the placebo group [32]. Abolfazl Mehdizadehkashi et al. showed that vitamin D intake led to a significant reduction in high-sensitivity C-reactive protein (hs-CRP) and a significant increase in total antioxidant capacity (TAC) compared with the placebo [24]. Another study showed that there was a significant decrease in peritoneal fluid inflammatory markers—RANTES (*P* ≤ 0.002), interleukin-6 (*P* ≤ 0.056), and monocyte chemotactic protein-1 (*P* ≤ 0.016)—after vitamins E and C therapy compared to patients not on vitamins [29]. However, one study suggested that after 2 months of vitamin C treatment, oxidative stress markers were unaffected in infertile endometriosis.

### Fertility

Regarding the effect of vitamin supplementation on pregnancy in women with endometriosis, there were only two articles available (Table 4). One study showed no statistically significant difference in the pregnancy rate between the supplementation group and the placebo group. Xiang Lu et al. suggested that after 2 months of vitamin C treatment, the retrieved oocyte rate, implantation rate, and clinical pregnancy rate were unaffected but could ameliorate the quality of oocytes and embryos.

### Risk of bias

**Fig. 7:**
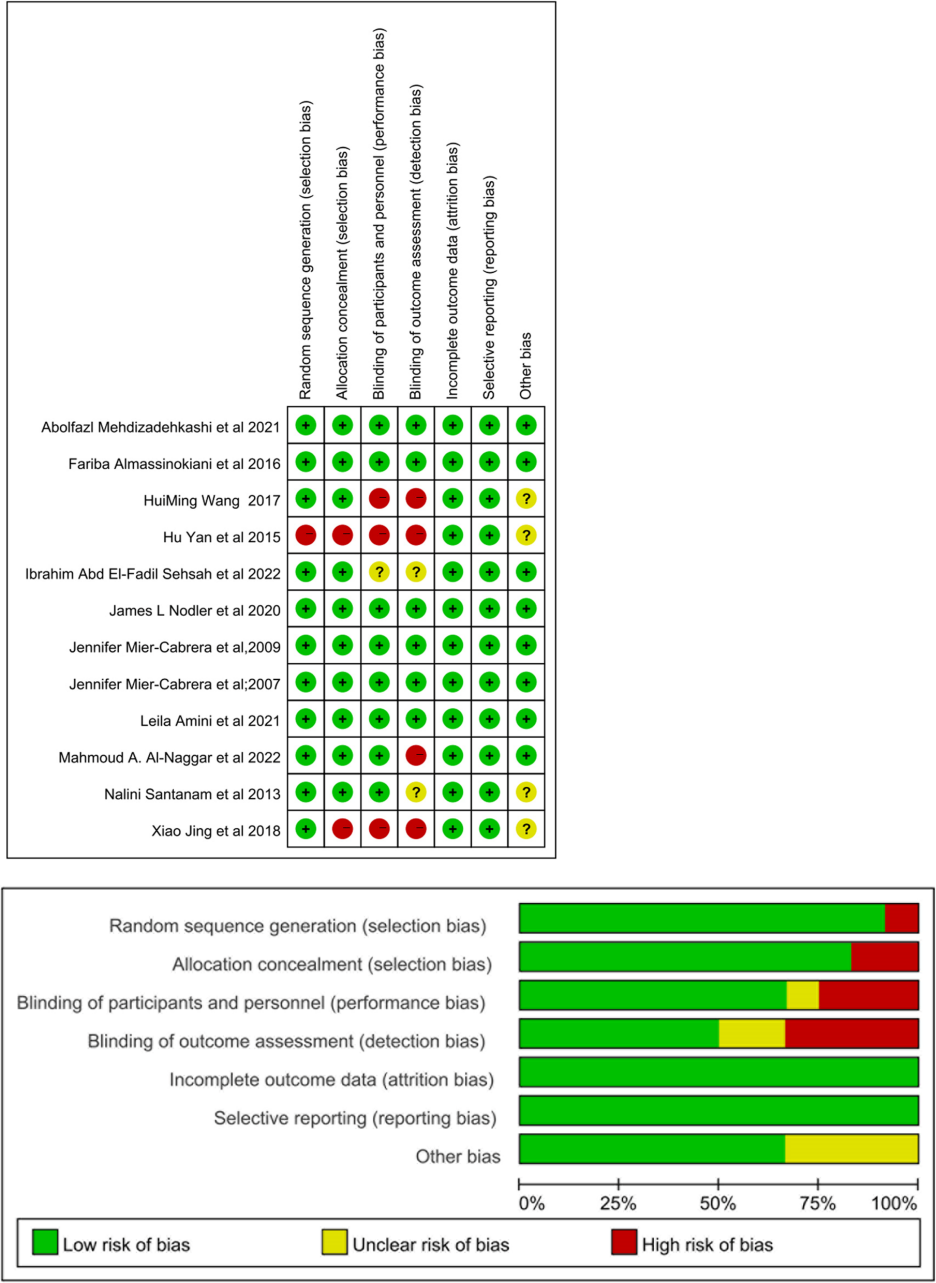
Risk of bias for RCTs

**Fig. 8:**
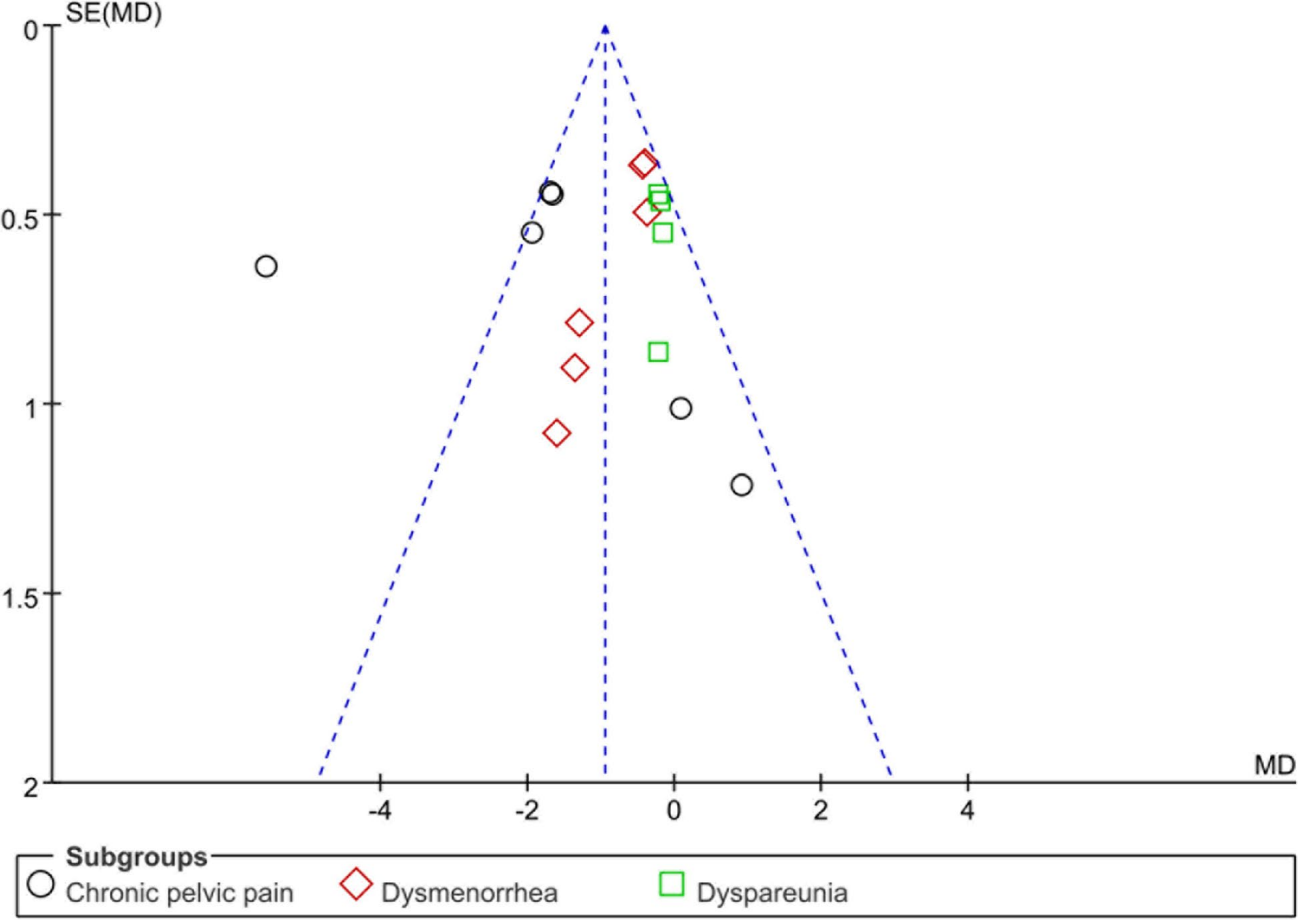
Risk of publication bias: funnel plot

The risk of bias assessments for RCTs is shown in Fig. 7. Among the RCTs, the highest risk of bias was observed in random sequence generation, allocation concealment, and blinding of participants and personnel. A funnel plot was used to assess publication bias in Fig. 8. The funnel plot and the Egger’s test (*p* = 0.173) did not suggest the presence of publication bias.

## Discussion

Reducing the symptoms of endometriosis, especially chronic pelvic pain, dysmenorrhea, and dyspareunia, is crucial for improving the physical and mental health of individuals with the disease. Current pain management approaches involve medications and surgical treatments, but their side effects and risk of recurrence have led to the exploration of alternative options. Could vitamin supplementation be a potential new treatment for endometriosis, specifically for reducing pain?

To examine the relationship between vitamin supplements and endometriosis, we conducted the first meta-analysis of studies on this topic. Our systematic review and meta-analysis included 11 studies, comprising a total of 589 patients, that evaluated the effect of antioxidant vitamins supplementation on endometriosis-related pain. The results indicated that antioxidant vitamins supplementation can effectively alleviate endometriosis-related pain, which is consistent with the findings of a study by Sukan et al. [6]. However, it is important to interpret our findings within the context of the study design, as there was heterogeneity in the treatment effect. This heterogeneity could be attributed to differences between studies in terms of design, interventions, doses, duration of administration, and outcome measures. To address some of this heterogeneity, we conducted various statistical analyses, such as subgroup analysis, prediction interval, and sequential removal of studies. In subgroup analyses (Supplementary table 1), for chronic pelvic pain, vitamin E supplementation, with or without vitamin C, demonstrated a positive effect on improving clinical pelvic pain. On the other hand, vitamin D supplementation showed a reduction in pelvic pain in endometriosis patients, but the difference compared to placebo was not significant. Heterogeneity among the included studies remained high, but its reduction from 88 to 49% was observed upon removing the study by Leila Amini et al. (2007) (Supplementary table 2). For dysmenorrhea, various types of vitamin supplementation were significantly effective in reducing pain. In the case of dyspareunia, combined vitamin E and C supplementation showed significant relief, whereas there was no significant difference between placebo and vitamin D or only vitamin E supplementation for dyspareunia pain. These results indicate that different vitamin supplements do not consistently alleviate endometriosis pain. It is important to note that the confidence in these results is reduced due to the limited number of studies. However, the small number of research articles makes it impossible to conduct a meta-regression.

Endometriosis is associated with clinically significant symptoms in approximately 75–80% of individuals, with chronic pelvic pain being the most common and impactful symptom on patients' quality of life [40]. Pain in endometriosis can be nociceptive (including inflammatory), neuropathic, or a combination of both [41]. Aberrant inflammation and dysregulation of inflammatory factors play a major role in endometriosis-associated pain [42]. Macrophages, neurogenic inflammation, lipid peroxides, and pain-inducing prostaglandins have been identified as key contributors to the pathophysiology of endometriotic pain [12]. Oxidative stress, characterized by an imbalance between reactive oxygen species (ROS) and antioxidants, is also involved in endometriotic pain [11, 43]. Previous studies have found that endometriosis patients have lower serum levels of vitamins A, C, and E compared to control groups, potentially due to antioxidant use during oxidation reactions. Animal and human studies have shown that consuming fruits, vegetables, or antioxidant supplements can reduce markers of oxidative stress [6, 44, 45]. Antioxidant treatment, including vitamin E and vitamin C, can effectively downregulate or inhibit inflammatory markers, such as interleukins 1 and 6 and monocyte chemotactic protein-1, which may contribute to the release of pain-inducing molecules. Vitamin E also exerts anti-inflammatory effects by inhibiting prostaglandin E2 production from arachidonic acid through a decline in cyclooxygenase activity [46].

In our meta-analysis, we observed changes in oxidative stress markers following vitamin supplementation. Antioxidant vitamins supplementation was associated with a decrease in plasma malondialdehyde (MDA) concentration in women with endometriosis. The plasma levels of MDA were inversely proportional to the duration and dose of vitamin E and C supplementation. Studies by Mier-Cabrera et al. [15, 37] demonstrated that vitamins C and E supplementation resulted in a decrease in oxidative stress markers (MDA and lipid hydroperoxides) in women with endometriosis [15, 39]. Additionally, peritoneal fluid inflammatory markers (RANTES, interleukin-6, and monocyte chemotactic protein-1) were significantly reduced following antioxidant therapy compared to no antioxidant treatment [29]. Amini et al. [32] observed a significant decrease in MDA and reactive oxygen species (ROS) levels with the administration of vitamin C and E combination, but no change in total antioxidant capacity (TAC) levels compared to the placebo group. Vitamin D supplementation was found to reduce hs-CRP and TAC levels [24]. Animal studies further demonstrated the reduction of endometriosis development and peritoneal inflammation following vitamin administration [47–50]. In summary, our findings suggest that antioxidant vitamins may alleviate the pain experienced by individuals with endometriosis by reducing the inflammatory response. Furthermore, vitamin supplementation in women with endometriosis did not significantly impact reproductive outcomes, although it may have a positive effect on oocyte and embryo quality. Future research is necessary to explore this aspect further.

## Strengths and limitations

The strength of our study lies in the inclusion of a greater number of articles for analysis, leading to more positive results compared to the previous review. Furthermore, a substantial proportion of the included studies were high-quality randomized clinical trials. Additionally, we conducted subgroup analysis to elucidate the effects of different vitamin supplements on chronic pelvic pain, dysmenorrhea, and dyspareunia. We also examined the impact of vitamin supplements on oxidative stress markers. These findings contribute to a deeper understanding of the mechanism of action of vitamins in endometriosis. However, there are certain limitations that cannot be avoided. Firstly, the number of studies in our analysis is limited, and the sample sizes are small, potentially affecting the reliability of the results. Secondly, the different doses of vitamins used in the included articles may have influenced the outcome of the meta-analysis. Moreover, the small number of research articles makes it impossible to conduct a meta-regression. Additionally, confounding factors such as sun exposure habits and daily diet were not evaluated, which could have an impact on the analysis results. Finally, due to a lack of data, we were unable to assess the effects of vitamin supplementation on pregnancy outcomes.

## Conclusion

Our study demonstrates that the use of antioxidant vitamins supplementation is generally effective in reducing endometriosis-related pain and inflammatory markers. Consequently, this therapy can be considered as an alternative treatment either on its own or in combination with other methods for managing endometriosis-related pain. However, considering the limited sample size and quality of the studies, further research is needed to provide a clearer understanding of the role of antioxidant vitamins supplementation in women with endometriosis.We also propose a future meta-analysis when new and similar studies are added to the literature.

### Supplementary Information

**Additional file 1.**

**Additional file 2.**

**Additional file 3.**

**Additional file 4.**

## Data Availability

The datasets used in this study can be found in the full-text articles included in the systematic review and meta-analysis.
