# Supplementary material for: Antioxidant vitamins supplementation reduce endometriosis related pelvic pain in humans: a systematic review and meta-analysis
Source: Reprod Biol Endocrinol. 2023 Aug 29;21:79. doi: 10.1186/s12958-023-01126-1 (PMC10464024; doi:10.1186/s12958-023-01126-1)
Supplement: Supplementary file 1 — Additional file 1. [file 12958_2023_1126_MOESM1_ESM.docx]

**Supplementary Data File 1 – Search Methods**

A comprehensive search approach was used to locate published studies. All databases were searched from inception to 16 March 2023. No date or language restrictions were applied.

**PubMed**

1.endometriosis/

2."endometrio*".ab,kw,ti.

3.endometrioma.ab,kw,ti.

4.endometrium.ab,kw,ti.

5.antioxidant

1. Vitamin
2. Vitamin C
3. Vitamin E
4. Vitamin D
5. 25-OHD
6. “25(OH)D”
7. “25-hydroxyvitaminD”
8. #1 (((endometriosis[MeSH Terms]) OR (endometrio*[Title/Abstract])) OR (endometrioma[Title/Abstract])) OR (endometrium[Title/Abstract])
9. #2 (((((((antioxidant[Title/Abstract]) OR (Vitamin[Title/Abstract])) OR (Vitamin C[Title/Abstract])) OR (Vitamin E[Title/Abstract])) OR (Vitamin D[Title/Abstract])) OR (25-OHD[Title/Abstract])) OR (25(OH)D[Title/Abstract])) OR (25-hydroxyvitaminD[Title/Abstract])
10. #1 and #2

**Web of Science**

Indexes=SCI-EXPANDED, SSCI, A&HCI, CPCI-S, CPCI-SSH, ESCI Timespan=All years

1.1#(((TS=(endometriosis)) OR TS=(endometrio*)) OR TS=(endometrioma)) OR TS=(endometrium)

2.2#(((((((TS=(antioxidant)) OR TS=(Vitamin)) OR TS=(Vitamin C)) OR TS=(Vitamin E)) OR TS=(Vitamin D)) OR TS=(25-OHD)) OR TS=(25(OH)D)) OR TS=(25-hydroxyvitaminD)

3.1# and 2#

**Cochrane Library**

1.endometriosis/

2."endometrio*".ab,kw,ti.

3.endometrioma.ab,kw,ti.

4.endometrium.ab,kw,ti.

5.1 or 2 or 3 or 4

6.antioxidant

7.Vitamin

8.Vitamin C

9.Vitamin E

10.Vitamin D

11. 6 or 7 or 8 or 9 or 10

12. 5 and 11

**Scopus**

1."endometriosis” /

2."endometrio*".ab,kw,ti.

3."endometrioma” .ab,kw,ti.

4."endometrium” .ab,kw,ti.

5.1 or 2 or 3 or 4

6."antioxidant”

7."Vitamin”

8."Vitamin C”

9."Vitamin E”

10."Vitamin D”

11."25-OHD”

12. “25(OH)D”

13.“25-hydroxyvitaminD”

14. 6 or 7 or 8 or 9 or 10 or 11 or 12 or 13

15. 5 and 14 and batter*

China National Knowledge Infrastructure (CNK):Search in Chinese only.
