# Supplementary figures and images for: Antioxidant vitamins supplementation reduce endometriosis related pelvic pain in humans: a systematic review and meta-analysis
Source: Reprod Biol Endocrinol. 2023 Aug 29;21:79. doi: 10.1186/s12958-023-01126-1 (PMC10464024; doi:10.1186/s12958-023-01126-1)

For continuous data:


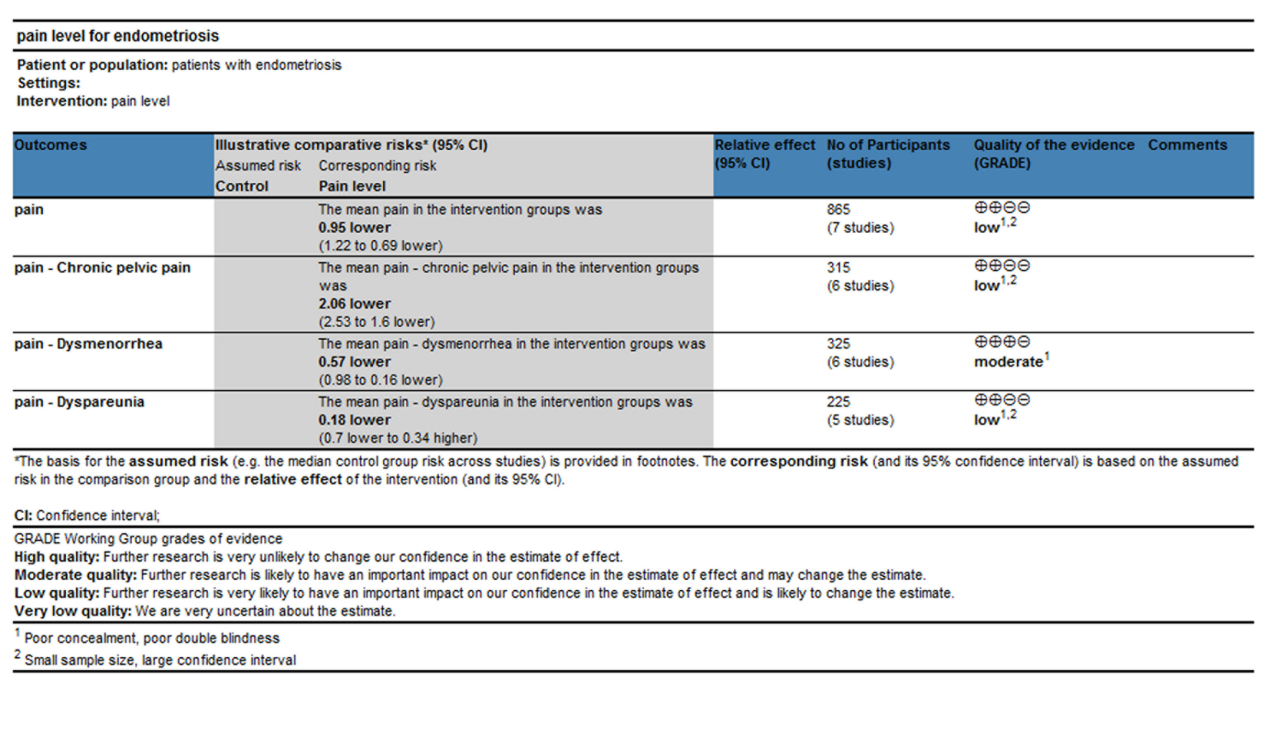


For categorical data


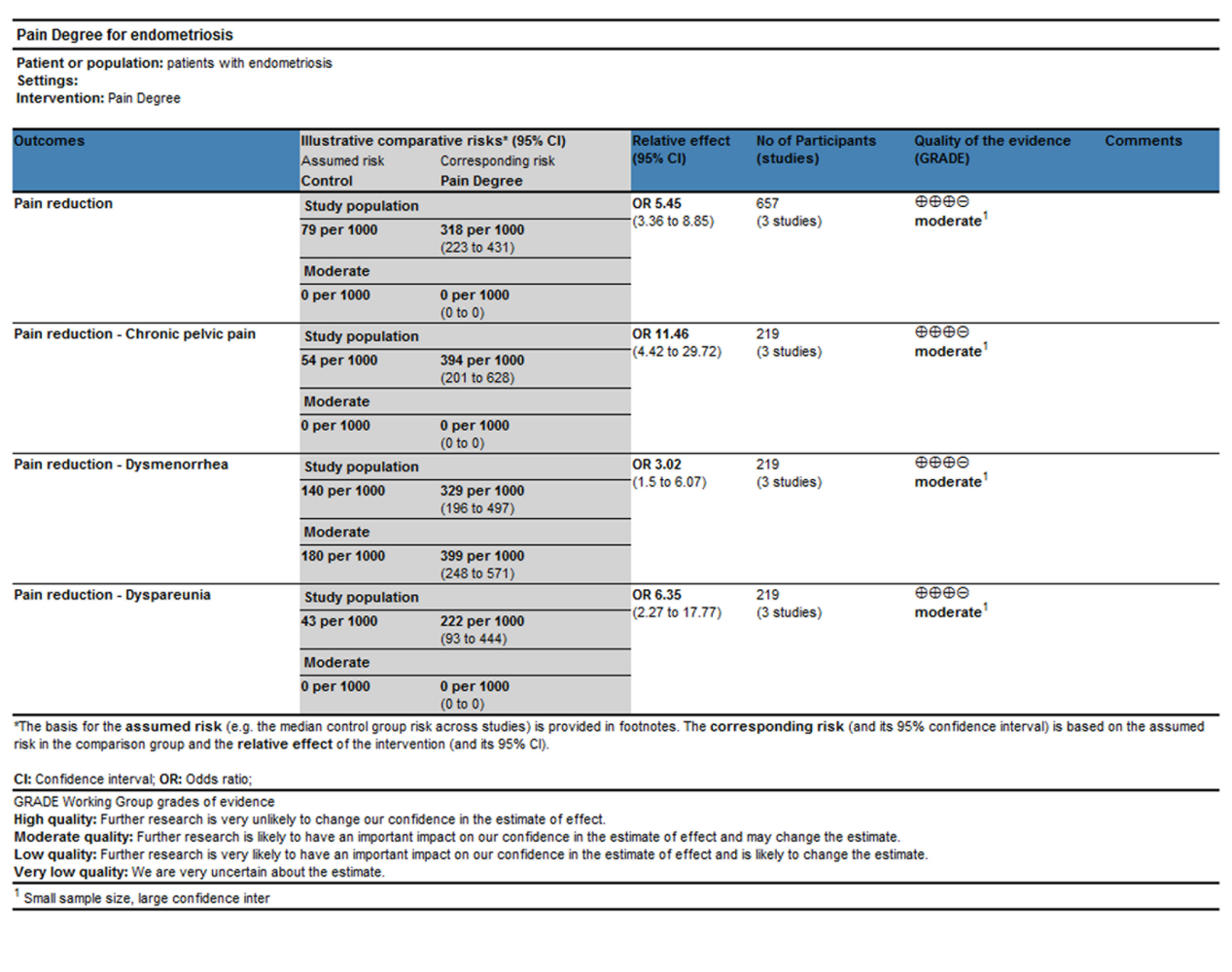

Supplement: Supplementary file 3 — Additional file 3. [file 12958_2023_1126_MOESM3_ESM.docx]
