# Supplementary material for: Antioxidant vitamins supplementation reduce endometriosis related pelvic pain in humans: a systematic review and meta-analysis
Source: Reprod Biol Endocrinol. 2023 Aug 29;21:79. doi: 10.1186/s12958-023-01126-1 (PMC10464024; doi:10.1186/s12958-023-01126-1)
Supplement: Supplementary file 4 — Additional file 4. [file 12958_2023_1126_MOESM4_ESM.docx]

Supplement Table1 . Various antioxidant Vitamins Supplementation and Endometriosis Related Pain(random effects).

CI, confifidence interva;

*---fixed effect mode

|  | Pain | Studies | Patients | I^2^ | Relative Risk(95% CI ) |
| --- | --- | --- | --- | --- | --- |
| Vitamin D | Chronic pelvic pain | 2 | 80 | 0% | 0.44(-1.08,1.96) |
|  | Dysmenorrhea | 2 | 80 | 0% | -1.40(-2.65,-0.15) |
|  | Dyspareunia* | 1 | 50 | NA | -0.2 (-1.9,1.5) |
| Vitamin E+C(continuous data)* | Chronic pelvic pain | 1 | 60 | NA | -5.54(-6.79,-4.29) |
|  | Dysmenorrhea | 1 | 60 | NA | -1.35(-3.12,0.42) |
|  | Dyspareunia | 1 | 60 | NA | -4.82(-6.22,-3.42) |
| Vitamin E+C(Categorical data)* | Chronic pelvic pain | 3 | 219 | 0% | 11.46(4.42,29.72) |
|  | Dysmenorrhea | 3 | 219 | 47% | 3.02(1.5,6.07) |
|  | Dyspareunia | 3 | 219 | 0% | 6.35(2.27,17.77) |
| VitaminE+Yasmin | Chronic pelvic pain | 3 | 175 | 0% | -1.73(-2.27,-1.20) |
|  | Dysmenorrhea | 3 | 175 | 0% | -0.41(-0.86,0.04) |
|  | Dyspareunia | 3 | 175 | 0% | -0.18(-0.73,0.36) |

Supplement Table 2 Prediction interval results with each study omitted

|  | SD | 95% CI | I^2^ | P-value | Chi^2^ | Df=5(P) |
| --- | --- | --- | --- | --- | --- | --- |
| Fariba Almassinokiani et al 2016 | -2.20 | (-3.69,-0.71) | 89% | 0.004 | 35.92 | <0.00001 |
| James L Nodler et al 2020 | -2.12 | (-3.67,-0.56) | 89% | 0.008 | 37.36 | <0.00001 |
| Leila Amini et al 2021 | -1.32 | (-2.07,-0.57) | 46% | 0.12 | 7.39 | 0.12 |
| Hu Yan et al 2015 | -1.72 | (-3.56,0.11) | 91% | 0.07 | 42.15 | <0.00001 |
| HuiMing Wang 2017 | -1.77 | (-3.68,0.14) | 90% | 0.07 | 41.04 | <0.00001 |
| Xiao Jing et al 2018 | -1.76 | (-3.68,0.16) | 90% | 0.07 | 41.22 | <0.00001 |
